# Expression and clinical significance of complement C3, complement C4b1 and apolipoprotein E in pancreatic cancer

**DOI:** 10.3892/ol.2013.1326

**Published:** 2013-04-30

**Authors:** JIONG CHEN, WEN WU, CHUNSHENG ZHEN, HANGCHEN ZHOU, RENBAO YANG, LONGJIANG CHEN, LIWEI HU

**Affiliations:** Department of General Surgery, Anhui Provincial Hospital Affiliated to Anhui Medical University, Hefei, Anhui 230001, P.R. China

**Keywords:** pancreatic cancer, complement C3, complement C4b1, apolipoprotein E

## Abstract

Pancreatic cancer (PC) remains a devastating disease with a five-year survival rate of <5%. The difficulty in making an early diagnosis and the frequent occurrence of metastasis are important reasons for this poor prognosis. In China, the incidence of PC has been increasing steadily. Therefore, the present study aimed to identify effective markers in the early and advanced stages of PC. The expression levels of complement C3, complement C4b1 and apolipoprotein E (ApoE) in the various stages of PC were assessed by immunohistochemistry, RT-PCR and western blotting. Additionally, the statistical significance of the results was analyzed. The expression levels of complement C3, complement C4b1 and apoE were higher in PC compared with normal pancreatic tissues. No correlations were observed between complement C3 and tumor TNM staging or lymph node metastasis. However, complement C4b1 and apoE were markedly correlated with tumor TNM staging and lymph node metastasis. Complement C3 may be used as a marker for the diagnosis of early-stage PC, while complement C4b1 and apoE are closely correlated with tumor development, reflecting the biological behavior of PC, and thus may be used as diagnostic markers of advanced PC.

## Introduction

Pancreatic cancer (PC), which is highly malignant, is a relatively common malignancy. Early surgical resection is the only effective treatment. However, due to its deep location and lack of pronounced early symptoms and specific clinical manifestations, an early diagnosis is difficult to make. The rapid development, fast growth and frequent lymph node metastasis all contribute to the poor prognosis of PC. The majority of patients with PC are diagnosed when the disease has reached an advanced stage and only 1–3% of patients survive up to five years (1). Therefore, an early diagnosis and future clinical developmental research is important. Tumor markers are a considerable tool for early tumor detection and screening. Tumor markers help improve the diagnosis of early PC and promote the prognosis of PC. Carbohydrate antigen (CA) 19–9 is one of the most common clinically used tumor markers for PC; however, it lacks sufficient sensitivity and specificity in early diagnosis (2–4). Other tumor markers, including carcinoembryonic antigen (CEA) and CA125, are also being studied; however, not specifically for the early diagnosis of PC. Therefore, it is crucial to identify more sensitive and specific diagnostic tumor markers to screen high risk patients and improve diagnosis (5,6).

## Materials and methods

### Ethical permission

The study protocol and consent forms conformed to the Declaration of Helsinki and were approved by the Ethics Review Board (ERB) Committee of the Affiliated Provincial Hospital of Anhui Medical University (Hefei, Anhui, China). Informed consent was obtained from all patients.

### Patients and samples

Between May 2007 and May 2009, PC specimens from 38 cases that had complete medical records were collected from the Department of Pathology of the Affiliated Provincial Hospital of Anhui Medical University. These cases consisted of 18 males and 20 females, aged 16–81 years, with an average age of 60.5 years. The specimens were divided according to their histological grade. A total of 29 cases were highly-differentiated and nine cases were poorly-differentiated, while according to the International Union Against Cancer (UICC) staging of primary PC, there were seven stage I cases and 31 stage II–IV cases. There were 23 cases with lymph node metastasis and 15 without metastasis. These samples were used for the immunohistochemical analysis. Between May 2007 and May 2009, a further 20 fresh specimens of PC and 20 adjacent normal pancreatic tissues were collected, all of which were confirmed by their pathology. Among these 20 fresh specimens, there were eight males and 12 females, aged 16–76 years, with an average age of 60 years. These cases were also divided according to histological grade. There were 13 highly-differentiated cases and seven poorly-differentiated cases, while the UICC staging of the original onset of PC showed four stage I cases and 16 stage II–IV cases. There were 13 cases with lymph node metastasis and seven cases without metastasis. These specimens were used for the RT-PCR analysis. None of the patients received pre-operative adjuvant treatment.

### Immunohistochemistry

Immunohistochemical staining was performed on 4-mm sections of formalin-fixed paraffin-embedded samples using a standard avidin-biotin-peroxidase complex technique. Following deparaffinization with xylene and rehydration with serial gradient ethanol, the antigen was retrieved by heating the slides in 10 mM citrate buffer (pH 6.0) for 6 min in a microwave. The endogenous peroxidase was blocked with 0.3% hydrogen peroxide. The slides were subsequently incubated with a blocking protein (normal goat serum) for 10 min and primary antibody was added overnight at 4°C followed by rinsing. Antibodies against C3, C4b1 and apolipoprotein E (ApoE; Santa Cruz Biotechnology Inc., Santa Cruz, CA, USA) were used. The secondary biotinylated antibodies, PV6000 (C3), PV9003 (C4b1) and PV9003 (ApoE), were then applied for 30 min, followed by 30 min of incubation with streptavidin peroxidase (Dako LSAB + HRP kit; DAKO, Carpinteria, CA, USA). Subsequent to being rinsed, the slides were visualized with diaminobenzidine (DAB) chromogen solution (Dako) and counterstained with hematoxylin, followed by dehydration through graded ethanol and slide mounting. The control group was treated with phosphate-buffered saline (PBS) instead of the primary antibody. To ensure the specificity of the primary antibodies, consecutive tissue sections were incubated in the absence of the primary antibody. No immunostaining was detected in these sections, indicating the specificity of the primary antibodies used in this study.

### Reverse transcription (RT)-PCR

Total RNA (Invitrogen, Carlsbad, CA, USA) was isolated from the fresh tissue samples using TRIzol. Cellular RNA (1 *μ*g) was used to perform RT using a Promega (Madison, WI, USA) RT kit and Oligo (dT) primers (A3800). All primers (Table I) were purchased from Takara (Dalian, China). Samples were amplified with an Applied Biosystem PCR System (Foster City, CA, USA) for 35 cycles with the following conditions: denaturation at 95°C for 30 sec, annealing at 51°C for 30 sec and extension at 72°C for 30 sec. β-actin was used as a control.

### Western blot analysis

The tissues were washed twice with cold PBS, then incubated in cold lysis buffer (1% Nonidet P40, 0.1% SDS, 150 mM Tris, 50 U/ml aprotinin and 1 mmol/l PMSF; pH 7.4) for 20 min at 4°C. The cellular lysate was centrifuged for 2 min at 12,000 × g at 4°C. The supernatants were collected as total cellular proteins. Quantitative analysis of the content was performed by the Lowry method and an equal amount of total protein from each sample was loaded onto SDS-PAGE gels. Following electrophoresis, the separated proteins were transferred to polyvinylidene fluo-ride membranes (Bio Trace PVDF; Pall Corporation, Port Washington, NY, USA) by electroblotting. Subsequently, the membrane was blocked with Tris-buffered saline (TBS) containing 5% skimmed milk for 1 h at room temperature, followed by incubation with primary antibodies against human C3, C4b1 and ApoE/TBS solution at 4°C overnight. Subsequent to being washed, the membrane was incubated in HRP-linked secondary antibody/TBS solution for 1 h at room temperature. The western blotting reaction products with chemiluminescence reagents (SuperSignal-West Femto Trial kit; Pierce, Woburn, MA, USA) were visualized by radiography.

### Data analysis

All experiments were repeated at least three times and representative results are presented. Data are expressed as the mean ± standard deviation. P<0.05 was considered to indicate a statistically significant difference.

## Results

### Immunohistochemical analysis of C3, C4b1 and ApoE

**Figure 1.**
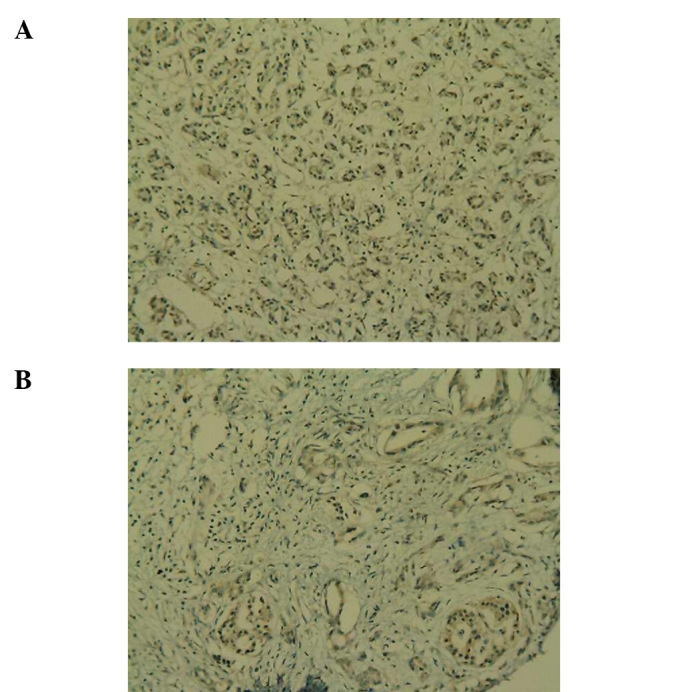
Immunohistochemical staining for C3. (A) Low C3 expression in normal pancreatic tissue. (B) High C3 expression in pancreatic cancer tissue (magnification, ×200).

**Figure 2.**
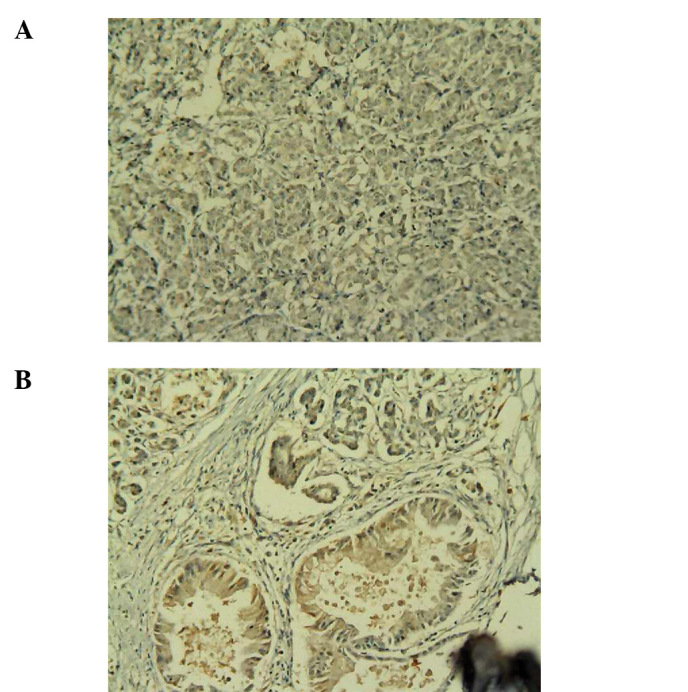
Immunohistochemical staining for C4b1. (A) Low C4b1 expression in normal pancreatic tissue. (B) High C4b1 expression in pancreatic cancer tissue (magnification, ×200).

**Figure 3.**
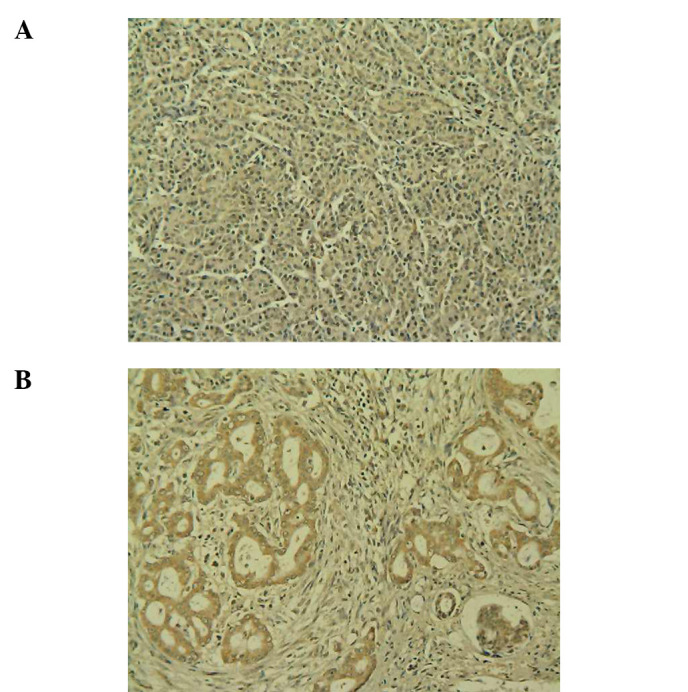
Immunohistochemical staining for ApoE. (A) Low ApoE expression in normal pancreatic tissue. (B) High ApoE expression in pancreatic cancer tissue (magnification, ×200). ApoE, apolipoprotein E.

The expression levels of C3, C4b1 and ApoE were significantly increased in the human PC samples (immunohistochemistry, magnification, ×200; Figs. 1–3). Weak positive C3 expression was observed in the normal human pancreas specimens (Fig. 1A), while positive C3 expression was observed in the PC specimens (Fig. 1B). Weak positive C4b1 expression was observed in the normal human pancreas tissues (Fig. 2A), while positive C4b1 expression was observed in the PC specimens (Fig. 2B). Weak positive ApoE expression was observed in the normal human pancreas tissues (Fig. 3A), while positive ApoE expression was observed in the PC specimens (Fig. 3B).

The immunohistochemical staining showed that the expression of C3, C4b1 and ApoE occurred in the cytoplasm. The C3, C4b1 and ApoE expression levels in PC and normal human pancreatic tissues were compared and the staining results showed that the human PC tissue had higher C3, C4b1 and ApoE expression levels (Table II).

In the human PC specimens, an average of 73.68 (28/38), 76.32 (29/38) and 86.84% (33/38) expressed C3, C4b1 and ApoE, respectively, whereas only 42.11 (16/38), 26.32 (10/38) and 42.11% (16/38) of normal human pancreatic specimens expressed C3, C4b1 and ApoE, respectively (χ^2^=7.77, 19.01 and 16.6, respectively; P<0.01).

Of the stage I PC specimens, 57.14% (4/7) expressed C3, while the rate of positive expression was 77.42% (24/31) in stage II–IV PC (P=0.19). In the PC specimens with lymphatic metastasis, 56.52% (13/23) expressed C3. The positive expression rate was 46.67% (7/15) in the PC tissues without lymphatic metastasis (P=0.74).

In the stage I PC specimens, 28.57 (2/7) and 57.14% (4/7) expressed C4b1 and ApoE, respectively, while the rates of positive expression were 87.10 (27/31) and 93.55% (29/31) in the stage II–IV PC specimens (P<0.05). In the PC tissues with lymphatic metastasis, 73.91 (17/23) and 78.26% (18/23) expressed C4b1 and ApoE, respectively, while the positive expression rates were 40.00 (6/15) and 33.33% (5/15) in the PC tissues without lymphatic metastasis (P<0.05).

### C3, C4b1 and ApoE mRNA expression in PC

**Figure 4.**
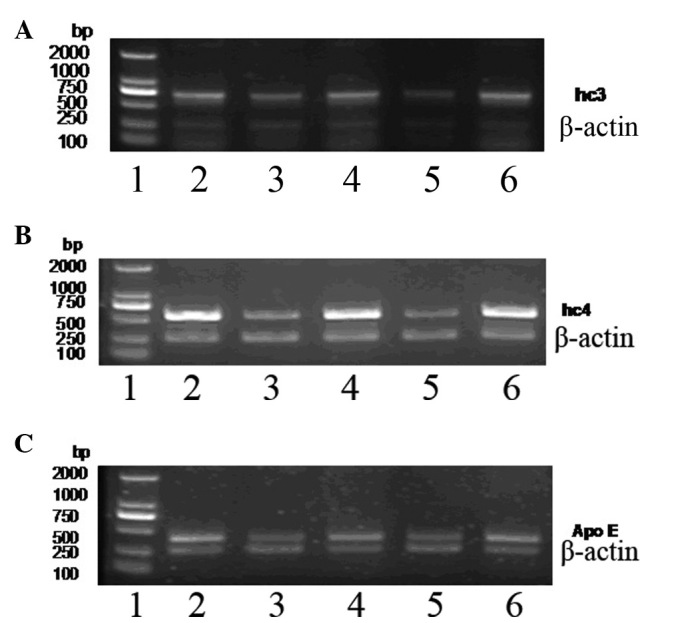
Expression of C3 (hc3), C4b1 (hc4) and ApoE mRNA. Lanes: (1) marker; (2) pancreatic cancer with lymph node metastasis; (3) stage I pancreatic cancer; (4) stage II–IV pancreatic cancer; (5) normal pancreatic tissue; and (6) pancreatic cancer without lymph node metastasis. ApoE, apolipoprotein E.

The expression levels of C3, C4b1 and ApoE in the PC specimens were determined by RT-PCR (Fig. 4).

The expression levels of C3 (5.93±0.82), C4b1 (7.94±0.95) and ApoE (4.83±0.65) mRNA in PC were significantly higher compared with the normal pancreas tissue levels of C3 (4.05±1.12; t=6.03, P<0.01), C4b1 (1.22±0.57; t=27.21, P<0.01) and ApoE (1.78±0.74; t=13.77, P<0.01; Table III).

In the stage I PC tissues, the expression level of C3 was 1.85±0.10, while in stage II–IV tissues, the expression level was 1.57±0.29 (t=1.85, P=0.08). In the PC specimens with lymphatic metastasis, the expression level of C3 was 1.56±0.31, while the expression level was 1.76±0.14 in the PC specimens without lymphatic metastasis (t=2.05, P=0.06; Table IV).

In the stage I PC tissues, the expression levels of C4b1 and ApoE were 1.11±0.08 and 2.32±0.03, respectively, while the levels were 1.28±0.19 and 2.63±0.20, respectively, in the stage II–IV PC samples (t=2.87 and 2.99, respectively; P<0.05). In the PC tissues with lymphatic metastasis, the expression levels of C4b1 and ApoE were 1.34±0.17 and 2.67±0.20, respectively, while the levels were 1.10±0.07 and 2.37±0.07, respectively, in the PC tissues without lymphatic metastasis (t=4.26 and 3.70, respectively; P<0.01; Table IV).

### Western blotting of C3, C4b1 and ApoE protein expression

**Figure 5.**
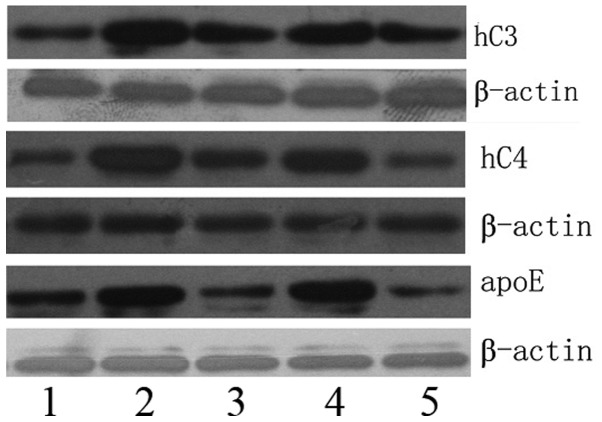
Western blotting of C3 (hC3), C4b1 (hC4) and ApoE protein expression. Lanes: (1) pancreatic cancer without lymph node metastasis; (2) pancreatic cancer with lymph node metastasis; (3) stage I pancreatic cancer; (4) stage II–IV pancreatic cancer; and (5) normal pancreatic tissue. ApoE, apolipoprotein E.

Western blotting was performed to determine the relative C3, C4b1 and ApoE protein expression levels in five pancreatic group types (Fig. 5). As expected, C3, C4b1 and ApoE were each detected as single bands. β-actin was used as a control. The C3, C4b1 and ApoE protein expression levels were higher in the PC samples and lower in the normal pancreas samples. Furthermore, the expression levels of C3, C4b1 and ApoE were observed to be significantly different in the various phases of PC (Table IV).

## Discussion

Complement is a sophisticated regulatory mechanism of the protein response system that is activated by antigen-antibody complexes or microorganisms and is involved in mediating inflammation, opsonophagocytosis, cell lysis, immune regulation and clearance of immune complexes. C3 is a mediatory inflammatory factor that, when activated, releases a variety of vasoactive substances (7,8), causing inflammation. In the present study, immunohistochemistry and western blotting demonstrated that the expression levels of C3 in the PC tissues were high, although the expression of C3 did not differ between the various stages of PC. The expression of C3 was not observed to be significantly different between the PC tissues with and without lymphatic metastasis. These findings indicated that C3 is involved in the early stages of PC, but not advanced PC. Therefore, C3 may be used as a serum biomarker in the early diagnosis of PC. The presence of C3, an important cytokine precursor involved in early PC, indicates that inflammation may be involved in the mechanism of PC. This is consistent with the study by Wang *et al* (9), which showed that the inflammatory regulator NF-κB was upregulated in PC tissues and cell lines. Certain studies have also reported that the expression of C3 is high in colon cancer serum (10), which may indicate that C3 is expressed specifically in the digestive tract.

C4b1 is not only closely associated with immune diseases, particularly systemic lupus erythematosus (SLE) (11), but it also has a crucial role in the induction of B cell self-tolerance (12). Theoretically, these are favorable factors for organisms fighting tumors, since antibodies bind to tumor antigens on tumor cells and are involved in directly killing them. Furthermore, immunoglobulin in B cell surfaces may combine with tumor antigens, inducing T cells to respond to tumors and creating antibodies, in collaboration with K cells, macrophages and complement, which are also able to kill the tumor cells [antibody-dependent cell-mediated cytotoxicity (ADCC)] and control tumor development and metastasis to a certain extent. Fujita *et al* analyzed the serum C4 levels of 43 cases of gastrectomy in gastric cancer patients (13). The results showed that higher levels of serum C4 were associated with higher recurrence rates. The present study also demonstrated that the expression of C4b1 in PC is high and markedly associated with the TNM stage of the tumor and with lymph node metastasis. The expression of C4b1 in the stage II–IV tumors and those with lymph node metastasis was significantly higher compared with the stage I tumors and those without lymph node metastasis, demonstrating that C4b1 is a marker of PC progression. We propose that the high expression levels of C4b1 in advanced PC may be the body’s protective immune response against the developmental process of PC. Therefore, this may generate new ideas for researching immune therapy for PC.

ApoE is a low-density lipoprotein (LDL) receptor ligand and its main biological function is the delivery of fat. ApoE is closely associated with lipoprotein metabolism, as well as the promotion of macrophage cholesterol overflow and the inhibition of platelet aggregation functions (14). The ApoE gene polymorphism produces the major human ApoE, generated in the liver and brain and other tissues, including mononuclear cells (such as macrophages). Between 60 and 80% of ApoE is synthesized in the liver. In addition, the human kidney, adrenal gland, bones and macrophages also synthesize ApoE. A previous study of ApoE was concerned with the association between ApoE and hyperlipidemia or related cardiovascular and cerebrovascular diseases (15), although in recent years, with more in-depth study, an increasing amount of research has been concerned with the role of ApoE in tumors. The expression of ApoE has been reported to be increased in a variety of tumors (16). Grant (17) reported that the upregulation of ApoE is a risk factor for prostate cancer. Andreotti *et al* (18) conducted a survey in Shanghai, China and showed that males with ApoE expression had an increased risk of suffering from bile duct cancer. The study by Moore *et al* (19) also revealed that ApoE and its gene variants increase the risk of renal cell cancer. Moreover, in the study of ApoE, the same close association was observed with gastrointestinal cancer (20). Mrkonjic *et al* (21) reported that diet (saturated fatty acid content) and ApoE isoforms synergistically increased the colorectal cancer risk. Grønborg *et al* (22) observed that the expression of ApoE in PC cells (Panc-1) was 20-fold higher compared with the normal control group. Yu *et al* (23) analyzed the expression of serum proteins in PC and normal controls by two-dimensional gel electrophoresis combined with a comparative analysis using tandem mass spectrometry and showed that the ApoE levels in the serum of patients with PC were significantly higher compared with the normal control group. Gillard *et al* (24) proposed that ApoE is distributed in the cells and is secreted by hepatic carcinoma cells. Huvila *et al* (25) demonstrated that ApoE has a positive correlation with endometrial adenocarcinoma and the degree of malignancy of the tumor. A higher expression of ApoE was associated with higher degrees of malignancy, indicating that ApoE reflects certain tumor biological characteristics. The present results showed that ApoE is highly expressed in PC. Furthermore, the ApoE levels were significantly higher in stage II–IV and lymph node metastatic PC tissues compared with stage I PC tissues and those without PC lymph node metastasis, thus showing that ApoE has an important role in the development of PC. This also indicates that ApoE reflects the biological characteristics of PC. The present results were consistent with those of Huvila *et al* (25). Martínez-Clemente *et al* (26) observed that ApoE was able to inhibit the expression of TNF-α, which is able to kill tumor cells. This also suggests that ApoE may be involved in tumor development. Additionally, the present results indicated that ApoE promotes the development of PC. Whether the mechanism is due to the inhibition of TNF-α remains to be clarified.

In conclusion, C3 may be used as a marker for the early diagnosis of PC. C4b1 and ApoE are closely correlated with tumor development, reflecting the biological behavior of PC, and may be used as diagnostic markers of advanced PC.

## Figures and Tables

**Table I.** Primers of C3, C4, ApoE and β-actin.

Gene	Forward primer	Reverse primer
C3	5′-TATCATCACCCCCAACATCT-3′	5′-CCCTCCACTTTCTTCCCGTA-3′
C4	5′-GGAAGCAAACGAGGACTAT-3′	5′-TTCAGCAGAACACAAGGTG-3′
ApoE	5′-CTGCGTTGCTGGTCA-3′	5′-GCTCCTCGGTGCTCT-3′
β-actin	5′-CAACTTCATCCACGTTCACC-3′	5′-GAA GAGCCAAGGACAGGTAC-3′

ApoE, apolipoprotein E.

**Table II.** Correlations of the expression levels of C3, C4b1 and ApoE with pancreatic cancer and pathological factors.

Factor	Category	No.	C3	C4b1	ApoE
Group	Pancreatic cancer	38	73.68% (28/38)	76.31% (29/38)	86.84% (33/38)
	Normal pancreas	38	42.11% (16/38)	26.32% (10/38)	42.11% (16/38)
	P-value		<0.01	<0.01	<0.01
UICC	Stage I	7	57.14% (4/7)	28.57% (2/7)	57.14% (4/7)
	Stage II-IV	31	77.42% (24/31)	87.10% (27/31)	93.55% (29/31)
	P-value		>0.05	<0.05	<0.05
Lymph nodes metastasis	No	15	46.67% (7/15)	40.00% (6/15)	33.33% (5/15)
	Yes	23	56.52% (13/23)	73.91% (17/23)	78.26% (18/23)
	P-value		>0.05	<0.05	<0.05

ApoE, apolipoprotein E; UICC, International Union Against Cancer.

**Table III.** Expression levels of C3, C4b1 and ApoE mRNA in pancreatic cancer.

Factor	Category	No.	C3	C4b1	ApoE
Group	Pancreatic cancer	20	5.93±0.82	7.94±0.95	4.83±0.65
	Normal pancreas	20	4.05±1.12	1.22±0.57	1.78±0.74
	t-value		6.03	27.21	13.77
	P-value		<0.01	<0.01	<0.01
UICC	Stage I	4	6.51±0.28	7.21±0.12	4.28±0.12
	Stage II–IV	16	5.78±0.85	9.14±1.02	5.28±0.81
	t-value		1.66	7.33	4.74
	P-value		>0.05	<0.01	<0.01
Lymph nodes metastasis	No	7	6.06±0.55	7.39±0.15	4.42±0.25
	Yes	13	5.86±0.95	8.24±1.07	5.05±0.71
	t-value		0.52	2.81	2.25
	P-value		>0.05	<0.05	<0.05

ApoE, apolipoprotein E; UICC, International Union Against Cancer.

**Table IV.** The GRAVY levels of C3, C4b1 and ApoE.

Factor	Category	No.	C3	C4b1	ApoE
Group	Pancreatic cancer	20	1.63±0.28	1.25±0.18	2.57±0.22
	Normal pancreas	20	0.88±0.19	0.65±0.13	1.28±0.24
	t-value		9.93	11.81	17.71
	P-value		<0.01	<0.01	<0.01
UICC	Stage I	4	1.85±0.10	1.11±0.08	2.32±0.03
	Stage II–IV	16	1.57±0.29	1.28±0.19	2.63±0.20
	t-value		1.85	2.87	<0.05
	P-value		>0.05	<0.05	2.99
Lymph nodes metastasis	No	7	1.76±0.14	1.10±0.07	2.37±0.07
	Yes	13	1.56±0.31	1.34±0.17	2.67±0.20
	t-value		2.05	4.26	3.70
	P-value		>0.05	<0.01	<0.01

GRAVY, grand average of hydropathicity; ApoE, apolipoprotein E; UICC, International Union Against Cancer.
